# The Putative Role of the Antiageing Protein Klotho in Cardiovascular and Renal Disease

**DOI:** 10.1155/2012/757469

**Published:** 2011-10-29

**Authors:** Giuseppe Maltese, Janaka Karalliedde

**Affiliations:** Cardiovascular Division, King's College London, 3rd Floor Franklin-Wilkins Building, Waterloo Campus, Stamford Street, London SE1 9NH, UK

## Abstract

Ageing is a multifactorial process often characterized by a progressive decline in physiological function(s). Ageing can and is often associated with an increased incidence of cardiovascular and renal disease. Klotho is a novel antiageing gene that encodes a protein with multiple pleiotropic functions including an emerging role in cardiorenal disease. Mice deficient for this gene display a phenotype of premature human ageing characterized by diffuse vascular calcification, altered calcium/phosphate metabolism, and shortened lifespan. Klotho is mainly expressed in the renal tubules but it also exists as circulating soluble form detectable in the blood, with systemic effects. Reduction in soluble Klotho has been associated with renal disease, hyperphosphataemia, increased oxidative stress, endothelial dysfunction, and diffuse vascular calcification. Conversely, overexpression of Klotho promotes cardiovascular-renal protection. The majority of the research on Klotho has been conducted in vitro and in animal studies but there is emerging data from human studies which suggest that Klotho may be a modifiable factor involved in the pathogenesis of cardiovascular and renal disease in at-risk populations. Further data is required to confirm if this novel protein can emerge as therapeutic tool that may be used to prevent or slow progression of cardiorenal disease.

## 1. Introduction 

Ageing is a complex phenomenon resulting from the interaction between genetic and environmental factors [1]. Advanced age is accompanied by higher prevalence of cardiovascular risk factors such as diabetes, hypertension, and chronic kidney that increase the risk of cardiovascular morbidity and mortality. All these risk factors are associated with endothelial dysfunction, which often precedes the development of overt disease [2]. Endothelial dysfunction is predominantly due to reduced availability of nitric oxide and increased oxidative stress which also promote the development and the progression of atherosclerosis and vascular calcification [3]. Vascular ageing is characterized by arteriosclerosis and calcification [4]. In conditions such as diabetes, there is premature vascular ageing which is associated with increased cardiovascular and renal disease risk. Further markers of vascular calcification appear to predict cardiovascular outcomes independently of conventional risk factors such as hyperlipidaemia, smoking, diabetes, hypertension, and family history of disease. The mechanisms that result in the development of vascular calcification are complex and have been reviewed in detail recently [5]. 

Klotho is a gene that encodes a novel protein regulating multiple functions, fortuitously discovered in 1997 by Kuro-o and colleagues and named after the goddess who spins the thread of life in Greek mythology [6]. 

In mice, the deletion of Klotho gene causes a phenotype of premature human aging including vascular calcification, altered calcium/phosphate metabolism with hyperphosphataemia, and shortened lifespan. 

Klotho protein exists in two forms: a type I transmembrane protein (1014 amino acids) with a large extracellular domain and a short intracellular portion (10 amino acids), predominantly expressed in the renal tubules, and a circulating soluble factor detectable in blood and in lesser extent in other biological fluids [7].

Soluble Klotho is produced either by proteolytic cleavage of the extracellular domain of the transmembrane form (130 kDa isoform) operated by the membrane-anchored proteases ADAM10 and ADAM17 or by alternative mRNA splicing (isoform 70 kDa) [7, 8]. The systemic effects of this protein appear to be predominantly due to the circulating form. The transmembrane protein forms a complex with fibroblast growth factor (FGF) receptors and works as an obligate coreceptor for FGF23, a bone-derived hormone that induces phosphate excretion into urine [9–11]. 

The fact that FGF23 requires Klotho for binding to its receptor explains why Klotho- and FGF23-deficient mice display identical phenotype [12, 13]. The observed hyperphosphataemia in Klotho and FGF23-mutant mice is due to hypervitaminosis D and increased expression/activity of renal sodium-dependent phosphate cotransporters. Interestingly, Klotho-deficient mice display higher levels of FGF23, and a low-phosphate diet reduces the levels of FGF23 and results in a rescue of the features of premature aging [13, 14]. This suggests that FGF23 per se cannot promote a phosphaturic effect in absence of Klotho [9].

Klotho/FGF23 signalling induces phosphaturia by suppressing the sodium-dependent phosphate cotransporters type IIa (NPT2a) expressed on the brush border membrane of renal tubular cells. Soluble Klotho has also been found to regulate directly the phosphate transport, in the proximal tubule of the kidney by deglycosylation of NaPi-2a cotransporters [15]. The resulting reduction in number and activity of NaPi-2a promotes phosphaturia independently of FGF-23. Soluble Klotho also inhibits type III sodium-dependent phosphate cotransporters (Pit1 and Pit2) which are ubiquitously expressed and mediate phosphate uptake [15].

High FGF23 levels in patients with chronic kidney disease are due to the declining renal clearance and also may represent a compensatory response to hyperphosphataemia [16].

Recent observational data suggest that FGF23 is associated with and may represent an independent risk factor for cardiovascular and all-cause mortality in patients with chronic kidney diseases stage of 4 and 5 (eGFR < 30 mL/min) [17, 18].

The reduction in Klotho expression observed in chronic kidney disease may be an important event contributing to the above with accumulation of FGF23 being a compensatory mechanism to the increase of phosphate levels driven by the primary reduction in Klotho. Recent data support this hypothesis as changes in Klotho levels appear to precede changes in phosphate levels, the key driver of FGF23 balance in renal disease [19].

## 2. Role of Klotho in Cardiovascular and Renal Disease

Klotho expression is affected by physiological and pathological factors. Renal expression of Klotho in rat is minimal in prenatal life but increases after birth [20]. A reduction in renal, serum, and urine levels of Klotho has been observed with normal ageing and in diseases characterised by premature vascular ageing such as renal disease as well as in animal models of disease such as diabetes and hypertension [21–24].  Table 1 summarises the conditions and disease states associated with reduction in Klotho levels. 

Conversely, there is evidence in animals that the overexpression of soluble Klotho can reverse the ageing process and provides cardiovascular-renal protection possibly by inducing resistance to oxidative stress and protecting tissues from oxidative damage [25, 26]. 

In this paper, we will summarize the key areas of research on the putative role of Klotho in prevention or delay of cardiorenal progression. We performed a Pub Med/Medline search for the terms Klotho, cardiovascular disease, and renal disease from 2000 to 2010 with a focus on recent mechanistic and proof-of-concept studies evaluating the role of Klotho in the prevention and treatment of cardiorenal disease.

## 3. Nephroprotective Effects of Klotho

Klotho is predominantly expressed in the renal distal tubular cells [6]. Animal studies have showed that the nephroprotective effects of this protein are mostly attributable to the antioxidant properties of its soluble form [27]. As outlined earlier, Klotho is a key mediator of phosphate balance in the nephron. 

Klotho expression is reduced in renal distal tubules, urine, and blood of rats subjected to bilateral renal ischemia [28]. Interestingly, the injection of an adenovirus harbouring the Klotho gene (which results in the release of soluble Klotho into the circulation) or the administration of recombinant soluble Klotho protein prior to the induction of the ischemic insult blunts the increase in creatinine and attenuates the tubulointerstitial damage [28–30]. Klotho expression is also downregulated in an animal model of spontaneous hypertension, and the delivery of Klotho has been shown to prevent the progression of hypertension, renal damage, and the proteinuria [31, 32]. Several mechanistic explanations for these observations have been proposed, and these centre on the reduction of renal superoxide and suppression of NADPH oxidase activity that is the main source of reactive oxygen species (ROS) which are all involved in the pathogenesis of renal disease. The observed nephro-protective effects appear to be at least in part independent of an acute (early) effect of Klotho on systemic blood pressure. However, treatment with Klotho does prevent the progression of spontaneous hypertension [32]. 

The nephroprotective effects of Klotho have also been tested in an animal model of glomerulonephritis [33]. The transgenic overexpression of Klotho in a mouse model of glomerulonephritis resulted in increased survival, attenuated glomerular and tubulointerstitial changes, and reduced proteinuria and blood urea nitrogen [33]. 

In vivo, the intraperitoneal administration of soluble Klotho recombinant protein, immediately after the induction of unilateral ureteral obstruction, prevents the acute renal fibrosis through the inhibition of TGF*β*1 signalling [34]. Specifically Klotho binds to the type II receptor (TGF*β*R2) suppressing the activation of the type I receptor (TGF*β*R1) that phosphorylates Smad2/3 proteins (transcription factors regulating the expression of TGF*β*1 target genes) [34].

Studies conducted in humans have reported a reduction of Klotho, both tissue (transmembrane) and soluble forms, in acute and chronic kidney disease. Koh et al. examined the kidneys of 10 patients with clinical or histological diagnosis of chronic kidney disease and demonstrated that the expression of Klotho protein was significantly reduced when compared to healthy control [35]. 

There are limited studies evaluating changes in serum and urine levels of Klotho in humans; Yamazaki and his colleagues were the first to establish a novel assay to detect circulating serum Klotho [24]. In 181 healthy Asian volunteers between 0.1 and 88 years of age, serum concentrations of Klotho ranged from 239 to 1266 pg/mL. Levels were higher in young subjects and lower in older adults and negatively correlated with serum creatinine levels [24].

A reduction of Klotho levels in urine has been reported in patients with acute kidney injury and in subjects with chronic kidney disease [28, 36]. Hu et al. have recently found in 39 patients with different severity of CKD lower levels of Klotho in urine. This decrease in urinary levels of Klotho is early at stage 1 and correlates with the decline of eGFR [36]. 

Klotho may be an early clinical biomarker of acute and chronic renal injury CKD as its diminution precedes changes of other well-established markers/factors involved in the progression of renal failure. However, further long-term prospective studies are required to establish the utility/value of Klotho as an early marker of acute and chronic renal disease.

## 4. Vascular Protective Effects of Klotho

Soluble Klotho has an important role in maintaining endothelial wall homeostasis and promoting the health of the vasculature [37–39]. In experimental models, the absence of Klotho gene is associated with endothelial dysfunction and diffuse vascular calcification [36, 38]. Recent experimental studies have confirmed that soluble Klotho may act as a humoral factor that protects the vascular system [39].

Endothelial dysfunction results from the imbalance between the release of vasodilator and vasoconstrictor factors and is an early step in the development and progression of cardiovascular disease. This disrupted equilibrium is predominantly due to the reduced bioavailability of nitric oxide (NO) because of its inactivation by ROS [3]. NO not only produces vasodilatation but also prevents the atherogenic mechanisms by suppressing smooth muscle cell proliferation and by inhibiting the expression of adhesion molecules and platelet aggregation [2]. 

Saito et al. demonstrated that in Klotho heterozygous mutant mice endothelium-dependent vasodilatation in the aorta and arterioles in response to acetylcholine is attenuated and the excretion of urinary nitric oxide metabolites is reduced [37]. In Klotho heterozygous mutant mice ischemia-induced angiogenesis is impaired and is accompanied by a decreased number of endothelial progenitor cells, which are important in the repair of damaged vessels, in the peripheral blood [40].

Klotho gene delivery, mediated by adenoviral vector, in a rat model of atherosclerosis, increased endothelium-dependent NO synthesis and prevented adverse vascular remodelling [37]. 

Klotho is also involved in the modulation of endothelial inflammation as demonstrated in vitro by Maekawa et al. In human umbilical vein endothelial cells (HUVECs), soluble recombinant protein might suppress the expression adhesion molecules involved in the pathogenesis of vascular disease such as intracellular adhesion molecule-1 (ICAM-1) and vascular cell adhesion molecule-1 (VCAM-1) [41]. 

Hu et al. have documented that Klotho is a direct inhibitor of vascular smooth muscle cell (VSMC) calcification. The authors showed in an elegant mouse model that Klotho deficiency is associated with more severe calcification, undetectable levels of soluble Klotho, and higher serum levels of phosphorus, whereas Klotho overexpression was accompanied by less calcification, preserved levels of Klotho and normal renal function [36]. In vitro, the recombinant soluble Klotho prevented the VSMC calcification induced by high phosphate through the inhibition of sodium-phosphate cotransporters Pit1 and Pit2 [36]. However, whether VSMC expresses endogenous Klotho is not known and this remains an important area for further research. In humans, the reduced urinary levels of Klotho in CKD might at least in part explain associated vascular calcification, a predictor of cardiovascular risk. As lack of soluble Klotho is an important factor in the pathogenic mechanisms of vascular calcification, its replacement may be a potential future therapeutic approach in the vascular risk management of patients with CKD [36].

## 5. Conclusions

**Figure 1:**
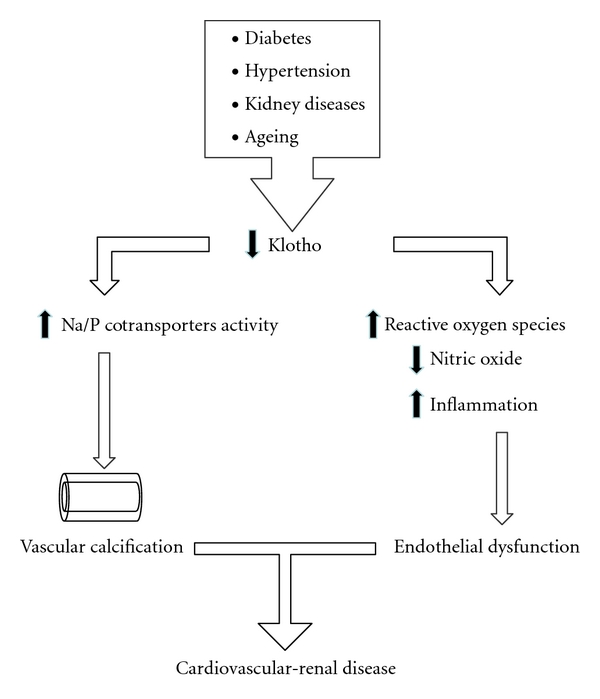
Putative mechanisms by which reduction in Klotho results in cardiorenal disease.

Soluble Klotho is a novel humoral factor that confers resistance to oxidative stress associated with ageing and several pathological conditions predisposing to cardiovascular-renal damage. There is emerging evidence highlighting the essential involvement of Klotho in calcium/phosphate metabolism and the maintenance of vascular integrity. Figure 1 summarises the potential mechanisms by which Kotho may afford cardiorenal protection. Since the decline in soluble Klotho levels represents a negative event occurring in the early stages of cardiovascular-renal disease, Klotho might be considered as a useful biomarker that predicts atherosclerosis and vascular calcification. Further long-term clinical studies are required to establish the role of this exciting new potential marker and predictor of cardiorenal disease.

## Figures and Tables

**Table 1:** Conditions and disease states associated with reduction in Klotho.

Disease/condition	Species
Ageing	Mouse/human
Diabetes	Mouse
Hypertension	Rat
Chronic kidney disease	Mouse/human
Acute kidney injury	Human
Kidney ischemia	Mouse
Glomerulonephritis	Mouse

## References

[1] Kirkwood TBL, Austad SN (2000). Why do we age?. *Nature*.

[2] Quyyumi AA (1998). Endothelial function in health and disease: new insights into the genesis of cardiovascular disease. *American Journal of Medicine*.

[3] Münzel T, Sinning C, Post F, Warnholtz A, Schulz E (2008). Pathophysiology, diagnosis and prognostic implications of endothelial dysfunction. *Annals of Medicine*.

[4] Lakatta EG (2003). Arterial and cardiac aging: major shareholders in cardiovascular disease enterprises. Part III: cellular and molecular clues to heart and arterial aging. *Circulation*.

[5] Liu Y, Shanahan CM (2011). Signalling pathways and vascular calcification. *Frontiers in Bioscience*.

[6] Kuro-o M, Matsumura Y, Aizawa H (1997). Mutation of the mouse Klotho gene leads to a syndrome resembling ageing. *Nature*.

[7] Matsumura Y, Aizawa H, Shiraki-Iida T, Nagai R, Kuro-o M, Nabeshima YI (1998). Identification of the human Klotho gene and its two transcripts encoding membrane and secreted Klotho protein. *Biochemical and Biophysical Research Communications*.

[8] Chen CD, Podvin S, Gillespie E, Leeman SE, Abraham CR (2007). Insulin stimulates the cleavage and release of the extracellular domain of Klotho by ADAM10 and ADAM17. *Proceedings of the National Academy of Sciences of the United States of America*.

[9] Kurosu H, Ogawa Y, Miyoshi M (2006). Regulation of fibroblast growth factor-23 signaling by Klotho. *Journal of Biological Chemistry*.

[10] Kuro-o M (2006). Klotho as a regulator of fibroblast growth factor signaling and phosphate/calcium metabolism. *Current Opinion in Nephrology and Hypertension*.

[11] Urakawa I, Yamazaki Y, Shimada T (2006). Klotho converts canonical FGF receptor into a specific receptor for FGF23. *Nature*.

[12] Shimada T, Kakitani M, Yamazaki Y (2004). Targeted ablation of Fgf23 demonstrates an essential physiological role of FGF23 in phosphate and vitamin D metabolism. *Journal of Clinical Investigation*.

[13] Razzaque MS, Sitara D, Taguchi T, St-Arnaud R, Lanske B (2006). Premature aging-like phenotype in fibroblast growth factor 23 null mice is a vitamin D-mediated process. *The FASEB Journal*.

[14] Stubbs JR, Liu S, Tang W (2007). Role of hyperphosphatemia and 1,25-dihydroxyvitamin D in vascular calcification and mortality in fibroblastic growth factor 23 null mice. *Journal of the American Society of Nephrology*.

[15] Hu MC, Shi M, Zhang J (2010). Klotho: a novel phosphaturic substance acting as an autocrine enzyme in the renal proximal tubule. *The FASEB Journal*.

[16] Gutierrez O, Isakova T, Rhee E (2005). Fibroblast growth factor-23 mitigates hyperphosphatemia but accentuates calcitriol deficiency in chronic kidney disease. *Journal of the American Society of Nephrology*.

[17] Gutiérrez OM, Mannstadt M, Isakova T (2008). Fibroblast growth factor 23 and mortality among patients undergoing hemodialysis. *The New England Journal of Medicine*.

[18] Jean G, Terrat JC, Vanel T (2009). High levels of serum fibroblast growth factor (FGF)-23 are associated with increased mortality in long haemodialysis patients. *Nephrology Dialysis Transplantation*.

[19] Razzaque MS (2009). FGF23-mediated regulation of systemic phosphate homeostasis: is Klotho an essential player?. *American Journal of Physiology*.

[20] Ohyama Y, Kurabayashi M, Masuda H (1998). Molecular cloning of rat Klotho cDNA: markedly decreased expression of Klotho by acute inflammatory stress. *Biochemical and Biophysical Research Communications*.

[21] Zuo Z, Lei H, Wang X, Wang Y, Sonntag W, Sun Z (2010). Aging-related kidney damage is associated with a decrease in Klotho expression and an increase in superoxide production. *Age*.

[22] Ohata Y, Arahori H, Namba N (2011). Circulating levels of soluble alpha-Klotho are markedly elevated in human umbilical cord blood. *Journal of Clinical Endocrinology Metabolism*.

[23] Semba RD, Cappola AR, Sun K (2011). Plasma Klotho and mortality risk in older community-dwelling adults. *The Journal of Gerontology Series A*.

[24] Yamazaki Y, Imura A, Urakawa I (2010). Establishment of sandwich ELISA for soluble alpha-Klotho measurement: age-dependent change of soluble alpha-Klotho levels in healthy subjects. *Biochemical and Biophysical Research Communications*.

[25] Kurosu H, Yamamoto M, Clark JD (2005). Physiology: suppression of aging in mice by the hormone Klotho. *Science*.

[26] Mitobe M, Yoshida T, Sugiura H, Shirota S, Tsuchiya K, Nihei H (2005). Oxidative stress decreases Klotho expression in a mouse kidney cell line. *Nephron Experimental Nephrology*.

[27] Yamamoto M, Clark JD, Pastor JV (2005). Regulation of oxidative stress by the anti-aging hormone Klotho. *Journal of Biological Chemistry*.

[28] Hu MC, Shi M, Zhang J, Quiones H, Kuro-o M, Moe OW (2010). Klotho deficiency is an early biomarker of renal ischemia-reperfusion injury and its replacement is protective. *Kidney International*.

[29] Sugiura H, Yoshida T, Tsuchiya K (2005). Klotho reduces apoptosis in experimental ischaemic acute renal failure. *Nephrology Dialysis Transplantation*.

[30] Sugiura H, Yoshida T, Mitobe M (2010). Klotho reduces apoptosis in experimental ischaemic acute kidney injury via HSP-70. *Nephrology Dialysis Transplantation*.

[31] Aizawa H, Saito Y, Nakamura T (1998). Downregulation of the Klotho gene in the kidney under sustained circulatory stress in rats. *Biochemical and Biophysical Research Communications*.

[32] Wang Y, Sun Z (2009). Klotho gene delivery prevents the progression of spontaneous hypertension and renal damage. *Hypertension*.

[33] Haruna Y, Kashihara N, Satoh M (2007). Amelioration of progressive renal injury by genetic manipulation of Klotho gene. *Proceedings of the National Academy of Sciences of the United States of America*.

[34] Doi S, Zou Y, Togao O (2011). Klotho inhibits transforming growth factor-*β*1 (TGF-*β*1) signaling and suppresses renal fibrosis and cancer metastasis in mice. *Journal of Biological Chemistry*.

[35] Koh N, Fujimori T, Nishiguchi S (2001). Severely reduced production of Klotho in human chronic renal failure kidney. *Biochemical and Biophysical Research Communications*.

[36] Hu MC, Shi M, Zhang J (2011). Klotho deficiency causes vascular calcification in chronic kidney disease. *Journal of the American Society of Nephrology*.

[37] Saito Y, Nakamura T, Ohyama Y (2000). In vivo Klotho gene delivery protects against endothelial dysfunction in multiple risk factor syndrome. *Biochemical and Biophysical Research Communications*.

[38] Nagai R, Saito Y, Ohyama Y (2000). Endothelial dysfunction in the Klotho mouse and downregulation of Klotho gene expression in various animal models of vascular and metabolic diseases. *Cellular and Molecular Life Sciences*.

[39] Shroff R, Shanahan CM (2011). Klotho: an elixir of youth for the vasculature?. *Journal of the American Society of Nephrology*.

[40] Shimada T, Takeshita Y, Murohara T (2004). Angiogenesis and vasculogenesis are impaired in the precocious-aging Klotho mouse. *Circulation*.

[41] Maekawa Y, Ishikawa K, Yasuda O (2009). Klotho suppresses TNF-*α*-induced expression of adhesion molecules in the endothelium and attenuates NF-*κ*B activation. *Endocrine*.

